# Few-Shot Emergency Siren Detection

**DOI:** 10.3390/s22124338

**Published:** 2022-06-08

**Authors:** Michela Cantarini, Leonardo Gabrielli, Stefano Squartini

**Affiliations:** Department of Information Engineering, Università Politecnica delle Marche, Via Brecce Bianche 12, 60131 Ancona, Italy; l.gabrielli@univpm.it (L.G.); s.squartini@univpm.it (S.S.)

**Keywords:** emergency siren detection, few-shot learning, prototypical networks, convolutional neural networks, domain adaptation

## Abstract

It is a well-established practice to build a robust system for sound event detection by training supervised deep learning models on large datasets, but audio data collection and labeling are often challenging and require large amounts of effort. This paper proposes a workflow based on few-shot metric learning for emergency siren detection performed in steps: prototypical networks are trained on publicly available sources or synthetic data in multiple combinations, and at inference time, the best knowledge learned in associating a sound with its class representation is transferred to identify ambulance sirens, given only a few instances for the prototype computation. Performance is evaluated on siren recordings acquired by sensors inside and outside the cabin of an equipped car, investigating the contribution of filtering techniques for background noise reduction. The results show the effectiveness of the proposed approach, achieving AUPRC scores equal to 0.86 and 0.91 in unfiltered and filtered conditions, respectively, outperforming a convolutional baseline model with and without fine-tuning for domain adaptation. Extensive experiments conducted on several recording sensor placements prove that few-shot learning is a reliable technique even in real-world scenarios and gives valuable insights for developing an in-car emergency vehicle detection system.

## 1. Introduction

Nowadays, although self-driving cars still represent a challenge, vehicles are equipped with driving automation such as cruise control, automatic steering, and parking assistance. Our focus lies on a novel emergency vehicle detection system [1,2,3], a safety device designed to alert the driver of emergency vehicles approaching, preventing distractions and siren perception difficulties due to cabin soundproofing or hearing impairment.

In recent years, research on emergency siren detection (ESD) has made significant improvements by combining sound signal processing techniques with deep neural networks. To mention some related works, in Ref. [4], a multi-task learning scheme has performed siren sound recognition taken from recordings and synthetic audio files. In Ref. [5], a multi-channel convolutional neural network (CNN) has executed the task of non-emergency and emergency sound classification. The authors have retrieved the siren audio files from the massive collection of labeled data called AudioSet [6], further extended by using data augmentation techniques. In Ref. [7], three neural network models based on 1D-CNN, 2D-CNN, and their ensemble have been developed for siren, vehicle horn, and noise classification. The dataset consists of thirty hours of audio files collected from web resources, publicly available data, microphone recordings, and data augmentation applications. In our previous works [8,9], we have achieved state-of-the-art performance in the ESD task by investigating several acoustic features and minimizing the computational cost of the algorithm. We have trained and tested a CNN with spectrograms computed from audio files equally balanced in siren and noise. For this purpose, we have downloaded noises from web resources and generated siren audio files via algorithm, collecting about thirteen hours of audio material.

Besides the neural architectures, the regularization and optimization techniques, and the performance achieved, most of the ESD works have in common a large amount of annotated audio data for training [10,11,12]. Typically, a large dataset represents the requirement of modern supervised deep learning models to build a robust system for detecting and classifying sound events [13,14,15], especially rare ones [16,17]. On the other hand, data are difficult to collect in some applications [18], and the manual labeling process involves human effort. A widely used method for extending small audio datasets consists of data augmentation procedures applied directly to the raw signal (e.g., noise addition, distortion, or speed scaling) or the time-frequency representation (e.g., pitch or time-shifting) [19,20]. However, these techniques have the disadvantage of altering the target signal or the background, creating criticality for detecting sounds with well-defined patterns and in specific contexts, such as an emergency siren in urban traffic noise. Therefore, with limited availability of annotated data and no recourse to data augmentation, the main goal is to devise detection systems that provide reliable results, given only few examples to build a neural model.

The purpose of the present study is to develop a deep learning-based siren detection algorithm for cars. For this task, we captured recordings onboard a vehicle equipped with microphones outside and inside the passenger compartment. The recording context has revealed the difficulty of capturing and labeling audio data containing siren sounds, the influence of sensor location in the signal acquisition, and the impact of car structural characteristics on sound quality. Collecting a large dataset of siren sounds is, thus, difficult and challenging. Given the scarce availability of labeled data containing siren sound events, in this work, we evaluate the effectiveness of few-shot learning applied to the emergency siren detection task, focusing on ambulance sirens.

Specifically, we investigate a few-shot metric learning method, i.e., prototypical networks in their original formulation [21]. Episodic training is performed in several configurations with three datasets extracted from publicly available sources and synthetic audio file collections. For each dataset, the model yielding the best results is applied, at inference time, to our noise and siren recordings, and lastly, the robustness of the prototypical technique is compared to a baseline model. For this purpose, we employ the convolutional architecture implemented in our previous work [8,9], trained with the synthetic dataset with and without fine-tuning for domain adaptation, and tested on our recordings. The experiments demonstrate that, in the condition of scarce availability of labeled data, few-shot methods return promising performance in emergency siren detection, outperforming traditional CNN models.

This work provides a comprehensive analysis of few-shot metric learning capabilities in a real-world application, where one of the most critical issues is the difficulty of collecting actual data as input to a deep learning algorithm. Our main contributions are summarized as follows.

1. We analyze the performance of prototypical networks employing datasets extracted from three audio collections differing in the genre, amount of data, and sound categories. In this step, we detail for what reason and to what extent the dataset characteristics and training/test examples combinations affect the performance of each prototypical model.
2. We propose the application of the knowledge learned by the few-shot models in discriminating similar or dissimilar instances from a specific audio collection to detect a target sound belonging to a different dataset (the ambulance siren recordings), given only few examples for the prototypical embeddings computation.
3. We focus the work on real-world applications, comparing the efficacy of different sensor placements onboard the vehicle. The analysis thus conducted provides relevant information to the development of emergency vehicle detection systems, such as the most suitable placement of the sound recording sensor between eight microphone positions outside and inside the cabin.
4. We validate the effectiveness of the few-shot technique by comparing it to a baseline with and without fine-tuning by using a few examples of the target domain. We also evaluate noise filtering strategies to improve the performance of the analyzed models.

The rest of the paper is organized as follows. Section 2 provides an overview of previous research on few-shot metric learning for audio and its relation to our study. In Section 3, we describe the proposed approach, and in Section 4, the datasets and the experimental configurations are presented. We illustrate and discuss simulation results in Section 5, and finally, we summarize the conclusion and comment on possible future extensions in Section 6.

## 2. Related Work

Although supervised learning with one or a limited number of examples has been a topic of interest for several years in computer vision [22,23], applications in audio signal processing are relatively recent. Among the different few-shot approaches [24,25], task-invariant embedding methods fit well with audio classification. The principle is that an embedding function is learned from a generic large-scale training set, and the prior knowledge in discriminating between similar and dissimilar instances is applied directly to new classes at inference time by using similarity measures.

This section summarizes some relevant work on few-shot deep metric learning in audio signal processing, paying particular attention to those that share the same few-shot techniques and methods employed in this work.

In acoustic event recognition and acoustic scene classification, [26] is the first work to address the challenge of deep learning with few audio data employing transfer learning strategies compared with prototypical networks [21], not in the concept of meta-learning but with a fixed taxonomy in training and testing. In Refs. [27,28], acoustic event detection with meta-learning models [21,29,30] shows the effectiveness of these approaches in generalization to new audio events, outperforming supervised solutions based on fine-tuned convolutional neural networks. Five different few-shot learning methods [21,31,32,33,34], improved with an attentional similarity module to detect transient events, are applied to sound event recognition in [35]. The effectiveness of few-shot techniques in sound event detection has led to the development of strategies to extend their application to increasingly challenging tasks, such as multi-label classification [36], rare sound event detection [37], continual learning [38], unsupervised and semi-supervised learning approaches [39], and sound localization [40].

Few-shot methods are adapted to an open-set sound event detection problem in Ref. [41], where several few-shot metric learning techniques are applied and compared to locate keywords in audio files. The task is transformed into binary classification, where the keywords belong to a positive set, and all other words constitute the negative set. The authors show that the method can generalize to unseen languages, and it could find applications to audio domains different from speech. The problem of open-set recognition coupled with few-shot learning is also faced in Ref. [42]. Two different autoencoder architectures with a multi-layer perceptron classifier are designed to identify target sound classes and reject unwanted ones. The dataset, consisting of sounds in domestic environments, has been created ad hoc by the authors and structured for few-shot learning applications [43].

Recently, few-shot neural networks have been deployed in bioacoustic event detection research. Audio recordings can be beneficial in monitoring the presence and behavior of some animals, but collecting and labeling a large training set of animal vocalizations are demanding and time-consuming tasks. This issue concerned Task 5 of the DCASE 2021 Challenge [44], where participants have improved over the prototypical baseline by applying several approaches, such as a transductive inference method [45], attention similarity and embedding propagation [46], data augmentation [47,48], segmentation [49], and the combination of the prototypical loss with knowledge distillation and attention transfer loss [50].

Several studies have employed few-shot techniques in audio applications. In Ref. [51], few-shot keyword spotting of new and user-defined words to be implemented in devices with a vocal interface is performed by prototypical networks with different temporally dilated CNN architectures. Sound anomaly detection of industrial machinery [52], speaker identification and activity recognition [53], automatic drum transcription [54], and underwater acoustic signal recognition in impulsive noise environments [55] are some exemplifying and not exhaustive few-shot learning applications for audio.

In this paper, few-shot emergency siren detection is performed by drawing inspiration from the most task-suitable methods available in the literature. The goals of the paper consist of:

- the investigation of a few-shot CNN-based architecture such as prototypical networks because of the low-computational cost and no setting limitations;
- the choice of training data not necessarily related to the task. In a car-related acquisition context, it is challenging to generate realistic simulated data that consider engine noise, cabin attenuation, and sound absorption due to the car interior and passengers. Consequently, we explore the performance of three datasets from Spoken Wikipedia Corpora [56], UrbanSound8K [57], and our A3Siren-Synthetic audio files collections;
- the adoption of an open-set procedure in testing, considering the problem as a binary classification siren vs. noise;
- the use of data from recordings in the actual testing environment directly at the inference stage. For prototypical networks, the availability of few labeled instances is not a limitation to generating the embedded prototypes of the target domain;
- the validation of the proposed approach by comparison with a baseline model and the study of performance improvement strategies, such as harmonic-percussive source separation techniques [58,59] that have previously shown their efficiency in the ESD task [9].

The benefits of a few-shot approach for emergency siren detection can find application in a feasible and customizable emergency vehicle detection system implementation in different vehicles.

## 3. Proposed Approach

This section presents our solution for emergency siren detection built on prototypical networks, compared with a CNN baseline model. We first illustrate the few-shot metric learning strategies adopted, then give an overview of the proposed ESD workflow, and finally describe the neural architectures employed in this work.

### 3.1. Few-Shot Metric Learning

Few-shot learning aims to solve a classification task given a target domain built on few examples. Hence, it is necessary to adopt strategies to create a model with generalization capability and quick adaptation to new domains. The few-shot metric learning approach usually employs a training set different from the test set. Training is performed in a *C-way K-shot* fashion, where *C* represents the number of classes (ways) and *K* the instances (shots) of each class employed in each iteration. This training method mimics the configurations that will arise at inference time, preferring large datasets and a high number of iterations to learn how to discern between different classes given only few input examples. The robustness of the model is evaluated with a metric-based function that returns the similarity measure between instances of the same class.

#### 3.1.1. Episodic Training

A common strategy in few-shot metric learning algorithms is the episodic training [32]. For this purpose, we have organized the training set in *F* folders, each containing a set of *M* labeled samples $T={\left\{\left(x_{1},y_{1}\right),\ldots ,\left(x_{m},y_{m}\right)\right\}}_{m=1}^{M}$. The feature vectors $x_{m}\in R^{D}$ have a fixed dimension *D*, and the labels $y_{m}\in \left\{1,\ldots ,L\right\}$ represent the *L* classes. A folder is selected in each iteration (episode), and a mini-batch of data is sampled randomly. A part of the mini-batch constitutes the support set, composed of C×K examples $S={\left\{\left(x_{1},y_{1}\right),\ldots ,\left(x_{i},y_{i}\right)\right\}}_{i=1}^{C\times K}$ where feature vectors $x_{i}\in R^{D}$ and labels $y_{i}\in \left\{1,\ldots ,C\right\}$, with C≤L. The remaining samples define the query set, composed of C×q examples $Q={\left\{\left(x_{1},y_{1}\right),\ldots ,\left(x_{j},y_{j}\right)\right\}}_{j=1}^{C\times q}$. The embedding function $f_{\phi }$ incorporates the support set *S* and query set *Q* into a lower-dimensional hypothesis space. Due to the reduction in tensor dimensionality and a meaningful representation in the transformed space, similar examples relative to the task are close, while dissimilar examples are easily differentiable. The metric function $g_{sim}$ performs the similarity measure between the support and the query set embeddings, hence the definition of few-shot metric learning. Different metric functions can be used, fixed, or with learnable parameters. The training is repeated until the minimization of the loss function $L_{\phi }$, representing the prediction error of the samples in *Q* conditioned by the comparison with the representation in *S*.

**Figure 1:**
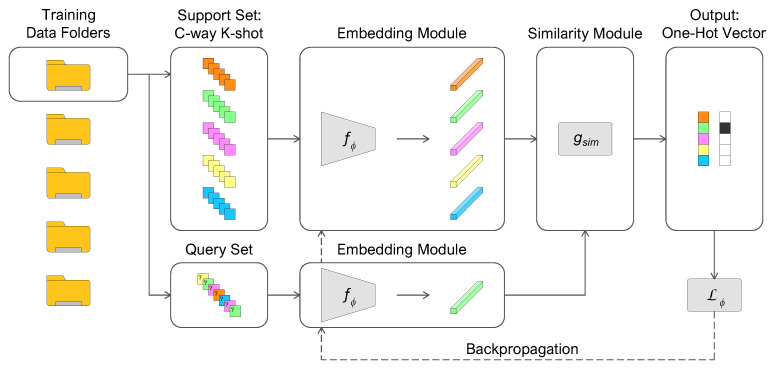
Episodic training procedure (*5-way 5-shot* example) of few-shot metric learning.

Figure 1 illustrates the episodic training procedure, from the random selection of support and query sets to the embeddings generation, and finally to the similarity measure in an iterative process to minimize the loss function.

#### 3.1.2. Open-Set Testing Procedure

The traditional few-shot testing method assumes that only U<F folders containing R<L classes are used in training. The embedding model must be optimized to transfer the knowledge learned to classify samples of the (L−R) classes in the (F−U) folders. Again, the support set *S* of size C×K and the query set *Q* with the instances to be classified are constructed. The problem thus posed is “closed-set” because samples belonging to *C* well-defined classes are classified.

Our work aims to detect samples of only one target class given few labeled instances for the embedding generation. Classifying samples of other categories, whose characteristics need not be necessarily known, is not of interest. The problem of discerning between one positive class and the negative rest is called “open-set” and is reduced to a binary classification task. At inference time, a positive support set $P={\left\{\left(x_{1},y_{pos}\right),\ldots ,\left(x_{i},y_{pos}\right)\right\}}_{i=1}^{p}$ consisting of a small number of labeled target samples and a negative support set $N={\left\{\left(x_{1},y_{neg}\right),\ldots ,\left(x_{j},y_{neg}\right)\right\}}_{j=1}^{n}$ containing examples that do not belong to the category of interest are randomly selected. The remaining instances compose the positive and negative query sets. As in training, the embedding function $f_{\phi }$ incorporates the support and query sets; the similarity module $g_{sim}$ compares the embeddings and returns the probability that the query sample belongs to the positive class. The algorithm must have generalization capabilities to find the similarity between the embeddings of the (unlabeled) target samples and those computed from the positive support set, discriminating from the negative class.

**Figure 2:**
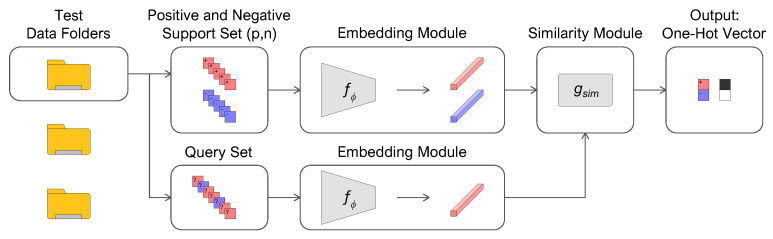
Open-set testing procedure of few-shot metric learning.

Figure 2 shows the open-set testing procedure, from the random selection of the positive and negative support sets to the embeddings generation, and finally to the similarity measure between the query and the positive support embeddings.

### 3.2. Overview of the ESD Workflow

The pipeline of the proposed approach consists of the following steps.

1. Best few-shot models computation: the raw audio is pre-processed and transformed into log-Mel spectrograms, organized, and given as input to prototypical networks. Because the episodic training in several *C-way K-shot* combinations returns different performance in the test phase, the model that gets the best output is saved for the next step. This procedure is repeated for three datasets extracted from diverse audio file collections, obtaining three best-performing *C-way K-shot* prototypical models.
2. Best few-shot models evaluation: our audio recordings are pre-processed and transformed into log-Mel spectrograms, split over eight audio channels corresponding to eight sensor positions. The best-performing *C-way K-shot* models obtained in step (1) are used to make predictions about new data taken from our recordings for the EDS task, repeating the procedure for each audio channel.
3. Analysis of prototypical outcomes: the experiments performed in step (2) provide classification scores distinguished by training model and recording channel, so we take the best performing dataset and the best sensor locations to compute the baseline models.
4. Baseline models computation: the raw audio belonging to the collection that provided the best prototypical results is pre-processed, and then log-Mel spectrograms are computed and organized in a suitable way as input to the CNN employed for the baseline. The network is trained in two ways: without domain adaptation and with fine-tuning by using combinations of few data taken from the target dataset.
5. Baseline models evaluation: we test the recordings on the baseline models computed in step (4), and the results are compared with few-shot best outcomes (step (3).
6. Harmonic filtered experiments: after applying a harmonic-percussive source separation technique, log-Mel spectrograms are extracted again from the recordings. The inference operations in steps (2) and (5) are repeated and compared to the experiments with unfiltered data.

**Figure 3:**
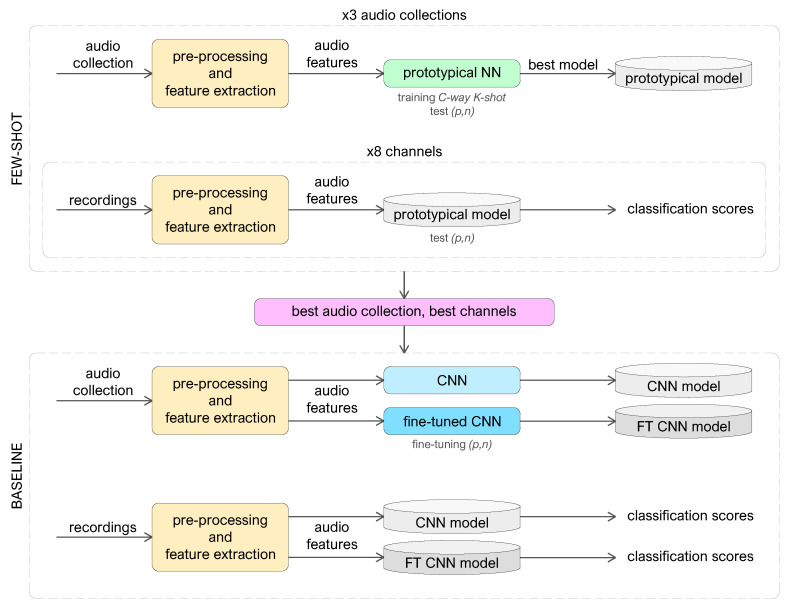
The block diagram of the proposed approach for emergency siren detection.

Figure 3 presents the block diagram of the proposed approach for emergency siren detection.

### 3.3. Neural Networks Architectures

#### 3.3.1. Prototypical Network

The peculiarity of a prototypical network is the generation of a representation $\mu_{c}$ of each class, called prototype. Given $S_{c}={\left\{\left(x_{1},y_{1}\right),\ldots ,\left(x_{c},y_{c}\right)\right\}}_{c=1}^{K}$ (the support set belonging to the *c*-class), the prototype $\mu_{c}$ is the mean vector of the embedded support samples, computed through the embedding function $f_{\phi }$ with learnable parameters ϕ:

(1) $$\mu_{c}=\frac{1}{K}\sum_{\left(x_{c},y_{c}\right)\in S_{c}}f_{\phi }\left(x_{c}\right).$$

Given a similarity function $g_{sim}$, represented by the squared Euclidean distance *d*, the prototypical network computes the relationship between a query sample $x_{q}\in Q$ and the prototypes via a softmax over distances in the embedding space:

(2) $$p_{\phi }\left(y=c|x_{q}\right)=\frac{\exp(-d\left(f_{\phi }\left(x_{q}\right),\mu_{c}\right))}{\sum_{c^{\prime }}\exp\left(-d\left(f_{\phi }\left(x_{q}\right),\mu_{c^{\prime }}\right)\right)},$$

where $p_{\phi }\left(y=c|x_{q}\right)$ represents the normalized probability distribution that $x_{q}$ belongs to the *c*-class. The training process is done by minimizing L(ϕ) (the negative log-probability of the true *c*-class via stochastic gradient descent):

(3) $$L\left(\phi \right)=-\log p_{\phi }\left(y=c|x_{q}\right).$$

Consequently, in each training episode, the model learns the similarity between query samples and the corresponding prototypes belonging to randomly chosen *C*-classes.

Prototypical architecture is based on the convolutional block defined in [33], consisting of a convolutional layer with a 3 × 3 kernel and 64 filters, a batch-normalization layer, and a ReLU activation layer. The sequence of four convolutional blocks, each followed by a 2 × 2 max-pooling layer, composes the embedding function $f_{\phi }$. In the learning process, the feature maps of the support set prototypes and the query set are flattened and concatenated to be compared through the similarity function $g_{sim}$.

#### 3.3.2. Convolutional Neural Network

The CNN architecture employed for the baseline consists of six convolutional layers and two fully connected layers [8,9]. The convolutional part is organized into three blocks with the same structure but a different number of filters. The first convolutional block comprises two convolutional layers with a 3 × 3 kernel and 4 filters, and an ELU activation function is applied after each of them. The final layer of each block performs a 2 × 2 max-pooling. The number of feature channels doubles in the subsequent two convolutional blocks, from 4 to 8 to 16. Then, the feature maps are flattened and given as input to a fully connected layer with 10 units. Finally, a softmax activation function is applied in the last layer.

## 4. Materials and Methods

This section first presents the audio file collections used in the experiments. Few-shot learning, whether based on meta-learning concepts or transfer learning techniques, exploits the knowledge learned in a source task and applies it in a different domain. For this reason, we experience non-task-related datasets in training, extracted from Spoken Wikipedia Corpora and UrbanSound8K. We also employ our audio files collection A3Siren-Synthetic, which has affinities with the test set computed from our recordings A3Siren-Recordings. Then, we describe the pre-processing operations on audio data, the features computation, the network training settings, and the performance metrics considered in the experiments.

### 4.1. Datasets

#### 4.1.1. Spoken Wikipedia Corpora

The Spoken Wikipedia Corpora (SWC) is an audio collection of volunteer readers of Wikipedia articles. The English-language corpus consists of 1339 audio files totaling about 395 h of recordings from various readers. The audio files, characterized by ogg format, are monophonic with a sampling rate of 44.1 kHz and 32-bit encoding. They are associated with metadata, some of which have textual annotations aligned to the words (start and end time in milliseconds). For the selection, partitioning, and pre-processing of the audio files, we have been inspired by the procedures described in [41].

In an SWC audio file, a specific word pronounced by a reader represents the target class. A *C-way K-shot* training episode is performed by taking a support set with *C* classes (different words) and *K* instances per class (examples of the same word) contained within a folder (corresponding to a reader). The query set is composed of an additional number of instances per class. We set up the episodic training with *C-way K-shot* ranging from *2-way 1-shot* to *10-way 10-shot* and a query set of 16 instances per class. Thus, we keep readers with at least 2 target words repeated 26 times, considering only audio files with temporally aligned words. Out of 208 readers and more than 2000 classes, we assign 75% to the training, 10% to the validation, and 15% to the test. Audio segments are selected by taking a 0.5 s window in the center of each instance. At inference time, the knowledge acquired to recognize the similarity between the same words spoken by a reader is transferred to detect a target word (positive set *p*) discriminating from various random samples of non-target words (negative set *n*) within an audio file of a reader assigned to the test.

#### 4.1.2. UrbanSound8K

UrbanSound8K (US8K) includes 8732 urban sounds of duration less than or equal to 4 s divided into 10 classes and 10 folders, totaling about 8.75 h. All audio files are in wav format, about 92% of which are stereo and the remaining 8% mono, with sampling rates ranging from 8 kHz to 192 kHz and encoding between 4-bit and 32-bit. The excerpts are taken from field recordings available at https://freesound.org/ (accessed on 5 June 2022) [60].

We split the training, validation, and test folders with a 7:1:2 ratio, leaving the distribution of the audio files within each folder unchanged and applying standardization operations. In a training episode, we select a folder (US8K fold) and take as support set *C* classes (environmental sounds) and *K* instances per class (examples of the same class also from different audio files) ranging from *2-way 1-shot* to *10-way 10-shot*. The query set consists of the remaining instances not used in the support set. We apply in testing the same positive and negative set definition criteria described for the SWC. In this case, training and testing are performed with a fixed taxonomy because, during the inference, we use classes already seen in training. It is not a limitation at this stage; our purpose is not to evaluate the performance of few-shot techniques on the US8K dataset but to apply different models to our recordings and compare the outcomes.

#### 4.1.3. A3Siren-Synthetic

A3Siren-Synthetic (A3S-Synth) is the audio collection used in our previous work [9] to train and test a convolutional neural network implemented for the siren vs. noise classification task. The training collection includes 32,000 noise and 32,000 siren audio segments in wav format, 16 kHz mono and 32-bit depth, each characterized by a 0.5 s duration. The noise examples were drawn from audio files downloaded from web resources [60], including urban traffic noise (car and motorcycle engines), and other varieties of sound events (horns, general alarms, heavy rain, people talking, and nature sounds) for about 4.5 h. On the other hand, the siren examples were generated via algorithm, according to the procedure described in [8]. Specifically, the Doppler effect was applied to an ambulance siren sound according to the Italian law [61]. Then, the implemented siren audio files were added to different environmental noises at SNRs equal to 0 dB, −5 dB, −10 dB, and −15 dB. We generated approximately 4.5 h of siren immersed in traffic noise to obtain a balanced dataset. The original test collection consists of 6000 noise and 6000 siren audio segments for each SNR used in training, having the same format and characteristics as the training set.

In this work, we carry out the few-shot experiments by organizing the training data into 16 folders, each containing 2000 noise and 2000 siren audio segments. Because only two classes are present, the episodic training is performed with *C-way K-shot* equal to *2-way 1-shot*, *2-way 5-shot*, and *2-way 10-shot*. The A3S-Synth training collection is split into 75% for training and 25% for validation. For evaluating the models, 300 noise and 300 siren audio segments for each SNR are randomly selected from the A3S-Synth test collection, organized in separate folders. We also employ this audio collection to train the CNN model representing the baseline as a comparison to the performance of few-shot techniques. In the experiments with the CNN, the organization of the original A3S-Synth training audio files is left unchanged. Again, the split is 75% for training and 25% for validation.

#### 4.1.4. A3Siren-Recordings

We call A3Siren-Recordings (A3S-Rec) the audio files recorded during a campaign conducted in May 2021. We used a car equipped with eight condenser microphones mod. Behringer ECM8000 (omnidirectional, impedance 200 Ohm, sensitivity 70 dB, frequency response 20–20,000 Hz, weight 136 g). The installation setup included four microphones inside the passenger compartment, with two at the sides of the front seats and two at the sides of the rear seats at seatback height, two in the trunk at the height of the floorboard, and two behind the license plate on opposite sides. The microphones have been connected via XLR connectors to an 8-channel Roland Octa-Capture soundboard, which in turn was interfaced via USB to a laptop controlled by an operator inside the car. The positions of the recording sensors have been designed to be distributed in all relevant places of the vehicle: at the front and rear of the passenger compartment, in the trunk, and externally. The positions inside the passenger compartment are evenly distributed within the cabin and do not interfere with the view, the air conditioning vents, or the audio system. In addition, they may have a use related to the audio equalization system [62]. The trunk represents a weather-protected environment scarcely affected by the sounds inside the passenger compartment and offers other sensor applications, such as asphalt wetness detection [63]. At last, the installation behind the license plate is a location in the outdoor setting that combines rapid responsiveness to external signals with a moderately sheltered condition from wind and weather.

**Figure 4:**
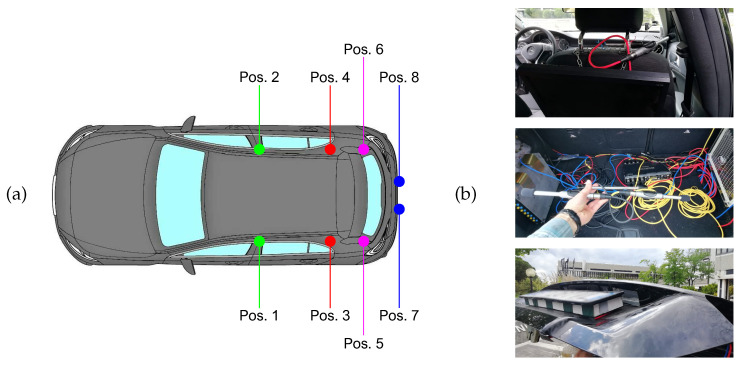
Setup of the car equipped for environmental recordings. (**a**) Layout of the microphone positions: positions 1–2 at the side of the front seats, positions 3–4 at the side of the rear seats, positions 5–6 in the trunk, and positions 7–8 behind the license plate. (**b**) Details of the microphones, from top to bottom: (top), at position 2; (middle), in the trunk at positions 5–6; (bottom), hidden behind the license plate at position 8.

Figure 4 shows the car’s microphone setup.

The recordings have been performed for a total of 7 days with the car moving in traffic and stopped at parking areas, always with the engine running. The itineraries have been carefully planned to cover the busiest roads where the transit of ambulances requires the activation of the siren alarm. We focused on the region’s most populated city, exploring high-traffic suburban areas near the main hospital and central urban areas. Sirens have been recorded in several situations: adjacent to a construction site, along a coastal road, in a suburban area near a shopping center and a residential neighborhood, in the city center, and on a high-speed road. In addition to various background noises, different driving settings have been requested based on the driving conditions: stationary with the engine running, at moderate speed with frequent stops in urban areas, and at high speed in suburban locations.

**Figure 5:**
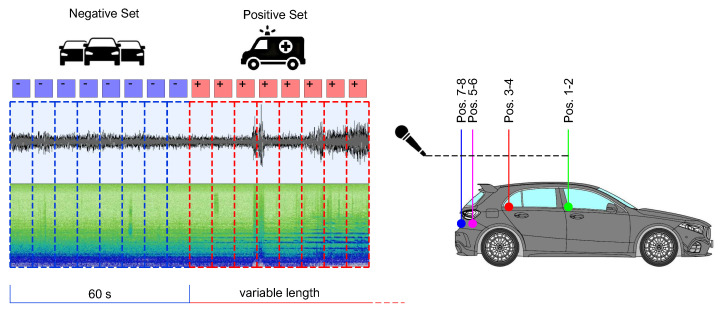
A3S-Rec positive and negative audio data selection method.

Recordings have been carried out separately in eight channels, corresponding to the eight microphones, with 44.1 kHz sampling rate and 32 bit-depth, and saved in wav format. We collected 18 audio tracks for approximately 10 h and 30 min. Only recordings containing siren events have been considered for the experiments, identifying six tracks for about 3 h and 39 min. The content of each track was analyzed, and the portion of the audio file in which the siren sound was audible, even weakly, with reference to the channels corresponding to the external microphones, was selected. Spectrogram visualization helped identify the presence of the fundamental frequencies and the upper harmonics of the two siren tones for the correct trimming of the audio files. After the standardization procedure application, 0.5 s siren segments have been assigned to the positive set. Given the wide availability of urban traffic noise, samples preceding each siren event for the duration of one minute have been chosen and attributed to the negative set. Figure 5 illustrates the proposed audio selection method, and Table 1 shows the A3Siren-Recordings composition.

We have organized the siren and noise audio segments in two ways: (i) in separate folders, each containing the samples selected in the individual recordings; and (ii) in a single folder in which all audio files are mixed regardless of the original track. The first strategy allows the algorithm to be evaluated in identifying the siren sound in a specific background noise context. In a single recording, the siren detection task is performed by contextualizing the target sound within a background noise where the siren gradually appears with a low signal level. The second data arrangement aims to discriminate between siren sounds and traffic noises not belonging to the same recording. In this way, we evaluate the capability of the network to recognize siren sounds in several background noises. In both cases, the siren detection is performed separately in the eight channels of the audio tracks.

### 4.2. Methods

#### 4.2.1. Audio Pre-Processing and Features Extraction

The audio files of each collection have different characteristics due to the recording devices and settings, so a pre-processing stage has been required to standardize them. The stereo audio files have been made monophonic through the operation of amplitude averaging of each audio channel; then they have been resampled to 16 kHz and encoded to 32-bit. Finally, they have been split into 0.5 s chunks with an overlap of 10 ms for sequenced segments. We chose log-Mel spectrograms as time-frequency representation. For each segment, log-Mel spectrograms represented by a 128 × 51 matrix have been computed on 25 ms Hanning windowed frames with a hop size of 10 ms, a 1024-point fast Fourier transform, and 128 Mel-frequency bands. Audio file pre-processing operations have been conducted with the Python libraries NumPy [64] for operations on arrays and LibRosa [65] for audio file loading, resampling, normalizing and writing. Feature extraction procedures have also been performed with LibRosa and Torchaudio [66] libraries.

#### 4.2.2. Harmonic Filtering

Our previous study [9] investigated the noise reduction and the siren harmonic components enhancement in the ESD task. Hence, the effectiveness of the harmonic-percussive source separation technique [58,59] is also evaluated in this work.

The procedure consists of the signal transformation from the amplitude to the frequency domain; then, horizontal and vertical median filters are applied to the spectrogram, resulting in one time-frequency representation with enhanced harmonic components and the other with accentuated percussive ones. The harmonic-percussive separation is also extended to a third residual component by drawing inspiration from the sines+transients+noise (STN) audio model [67]. According to this, the original spectrogram can be decomposed into three components:

(4) S=H+P+R,

where *H* contains the harmonic, *P* the percussive, and *R* the residual components not included in *H* or *P*.

The relationship between the harmonic and percussive contents (and vice versa) of the spectrograms obtained from the decomposition is regulated by two separation factors $(\beta_{h},\beta_{p})\geq 1$. In the experiments, we apply a harmonic separation factor $\beta_{h}=3$ margin coefficient of librosa [65] library) that accentuates the harmonic contents and reduces the percussive and residual ones.

#### 4.2.3. Training Settings

We train prototypical networks in several (C,K) configurations. In few-shot algorithms, it is not straightforward to design the validation process as an unbiased evaluation of the training fit, usually performed by a percentage of training samples at the end of each iteration. Therefore, we carry out episodic validation according to [33]: it is performed in the *C-way K-shot* fashion at the end of a chosen number of training episodes, employing validation folders split from the training ones. After each validation step, the average loss is compared with the previous value, and the learned parameters of the model that returned the best performance are stored for testing. We set the learning rate equal to 0.001, Adam [68] optimizer, and 60,000 episodes, of which 1000 for validation every 5000 for training, saving the model that performs best in the validation stage.

Then, the baseline model compared with the few-shot methods is computed with and without fine-tuning for domain adaptation. First, the CNN is trained for 100 epochs with a learning rate equal to 0.001, a decay rate of 0.1 every 30 epochs, and Adam optimizer, saving the model that performs best in validation. Then, the previously trained model is adapted with several (p,n) instances of the new task, according to the (p,n) combinations employed for prototypical support embeddings. Because we use few data for domain adaptation, the network is prone to quick overfitting. Hence, we freeze all the convolutional layers and re-train only the two last linear layers with a low learning rate and a small number of epochs. We set 20 epochs, Adam optimizer, learning rate between 0.0001 and 0.00001, and early stopping regulated by the training loss.

In our experiments, we used a NVIDIA DGX Station A100 with Dual 64-Core AMD EPYC 7742 @3.4 GHz, and 8 NVIDIA A100-SXM4-40 GB GPUs. The server was running Ubuntu 20.04.3 LTS, and we employed the PyTorch [69] deep learning framework to implement the network designs.

#### 4.2.4. Performance Metrics

Performance is evaluated in terms of the area under precision-recall curve (AUPRC), a metric used in binary classification with unbalanced data, focusing on positive examples. AUPRC scores range from 0 to 1. A model achieves a perfect AUPRC (equal to 1) when all true positive examples are correctly classified with neither false positives (perfect precision) nor false negatives (perfect recall). Both for prototypical networks and the fine-tuned baseline, the random selection of positive and negative instances could affect the results because of the variability of the sample characteristics, even within the same class. So, the experiments are repeated ten times with different random (p,n) examples, and the AUPRC scores are averaged to generalize the performance over the available data.

## 5. Experiments and Discussion

### 5.1. Few-Shot Models Analysis

The first step of our experiments aims to find the (C,K,p,n) combination that returns the best results in the classification of a target word (SWC), an environmental sound (US8K), and an ambulance siren (A3S-Synth). We train prototypical networks in the (C,K)∈{(2,1),(2,5),(5,1),(5,5),(10,1),(10,5),(10,10)} configurations with SWC and US8K datasets, while we employ (C,K)∈{(2,1),(2,5),(2,10)} with A3S-Synth. At inference time, all models are evaluated by constructing positive and negative support embeddings in the same (p,n) combinations with p∈{1,5} and n∈{1,5,10,50}. The results show the standard deviation across the AUPRC scores computed for each folder and averaged over 10 iterations.

**Figure 6:**
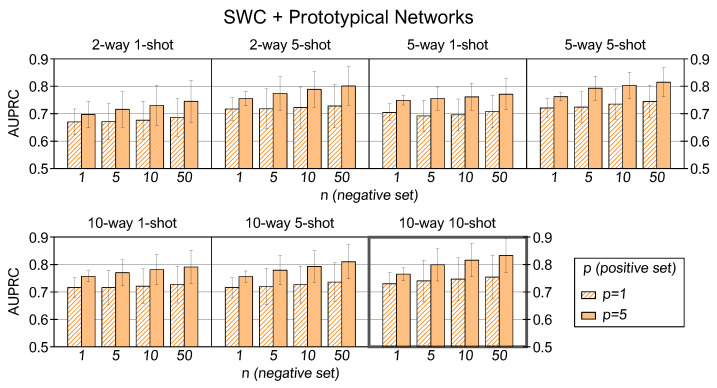
AUPRC results for prototypical networks trained and tested with SWC in several (C,K,p,n) combinations.

Figure 6 illustrates the performance of prototypical networks with the SWC dataset. The experimental results correlate better scores with higher (C,K,p,n). The *10-way 10-shot* model returns an AUPRC of 0.83 in the (p,n)=(5,50) combination and 0.77 averaged across all the (p,n) settings.

**Figure 7:**
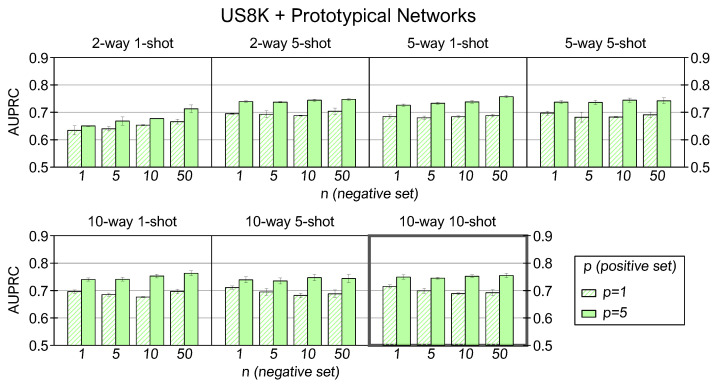
AUPRC results for prototypical networks trained and tested with US8K in several (C,K,p,n) combinations.

Figure 7 exposes the outcomes of prototypical networks using the US8K dataset. Again, the *10-way 10-shot* model yields the best results. The AUPRC for (p,n)=(5,50) is equal to 0.76, and the average score is 0.72 over all (p,n) combinations.

**Figure 8:**
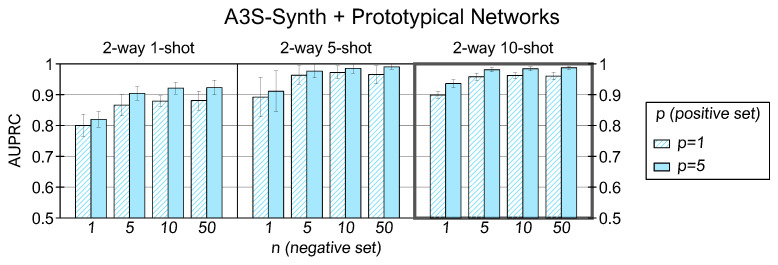
AUPRC results for prototypical networks trained and tested with A3S-Synth in several (C,K,p,n) combinations.

Figure 8 presents the prototypical results with the A3S-Synth dataset. Because *C* is fixed and equal to 2, we analyze the performance varying (K,p,n). Across the examined cases, the *2-way 10-shot* model returns the best score with an AUPRC equal to 0.99 employing (p,n)=(5,50) and 0.96 averaged over all the (p,n) combinations.

We now analyze the experimental results in each dataset varying (C,K,p,n). Optioning multiple values of *C* ways is allowed only in multiclass datasets, so we first examine the SWC and US8K experiments by fixing C=(2,5,10) and averaging the AUPRC scores over all the (K,p,n) corresponding configurations. In both datasets, we observe that many *C* ways improve the average scores: multiclass training expands the prior knowledge of the model by increasing the discriminative capability among many classes of sounds and facilitating the discernment between examples belonging to new classes at the inference stage. The *10-way* setting returns an average AUPRC equal to 0.76 for the SWC dataset and 0.72 for the US8K. One possible explanation for this lower performance is that the US8K comprises only ten classes, and few classes in training are limiting for constructing a model with high generalization capability.

We proceed to investigate the impact of different *K* values within the same *C-way* setting. For the SWC, many *K* shots return better performance: a higher number of examples creates a prototype that more closely collects the patterns of the original class, so the mean feature vector is more representative and facilitates the mapping between the positive queries and the corresponding support prototype. For the A3S-Synth, we observe that the *1-shot* condition is the least effective, and at the same time, there is no substantial improvement between the *5-shot* and *10-shot* settings. It is probably due to the low inter-class variance of support examples and the significant background noise level in the audio files, which could penalize the prototype generation with many examples. Finally, the dataset that shows the least benefit in using multiple *K* shots in the training prototype generation is the US8K. Again, the low interclass variability might invalidate the creation of representative prototypes with numerous examples, especially for stationary sounds such as those belonging to the air conditioner, drilling, engine idling, and jackhammer classes. In addition, other sounds might not be adequately represented by a 0.5-s time window, which is too wide for gunshot or too narrow for street music or children playing classes.

The outcomes of individual *C-way K-shot* cases by varying (p,n) are now explored. For all datasets and in all the (C,K,n) configurations, the performance of prototypical networks with p=5 is better than p=1 because the prototype created by one example is not always representative of an entire class. On the other hand, increasing *n* does not provide univocal results for all datasets. With the SWC and A3S-Synth, we notice an improvement in AUPRC scores as *n* increases, and the n=50 case returns the best results in all simulation contexts. Hence, using more examples to create the negative support prototype enhances the capability of the network to classify the positive instances correctly. However, this is not the case with the US8K dataset, which shows a similarity between *n* at inference time and *K* in the training phase, where the use of an increasing number of shots does not produce a more robust prototypical representation.

### 5.2. Siren Detection with Prototypical Networks

#### 5.2.1. Evaluation within Individual Recordings

This first analysis assesses the ESD task within individual recordings composed of audio segments with only traffic noise and others with additional sirens gradually arising from the background. In this way, we test the models to promptly identify the target sound in contexts where the background noise is significant, variable, and unpredictable. In the experiments, we separately analyze the performance of the recording sensors for each microphone position inside and outside the passenger compartment to evaluate which setup provides the best response.

Table 2, Table 3 and Table 4 present the results of the best prototypical models trained with the SWC, US8K, and A3S-Synth datasets, respectively, and tested on the single audio tracks of the A3S-Rec dataset for each acquisition sensor. We analyze several (p,n) configurations with p∈{1,5,10} and n∈{1,5,10,50} support examples. AUPRC is the average of the scores calculated for the six individual recordings over 10 iterations with random (p,n) support instances. Compared to the previous experiments for selecting the most performing models, we also investigate the combination with p=10 as the three datasets have returned the best results in the *10-shot* condition.

The results obtained by varying (p,n) for each training dataset and audio recording channel are now discussed.

With *n* fixed and *p* variable, we observe that the three models provide outcomes with the same trend in all audio channels: p=1 offers the worst performance and p=(5,10) shows improvements. In most cases, the AUPRC scores increase along with p=(1,5,10), although there are configurations in which p=5 equals or exceeds p=10 in performance. The smallest gap between the results with p=5 and p=10 can be observed in the channels 5–8. The reason is attributable to the noise in the recordings acquired inside the trunk and behind the license plate: using more examples does not always help create a more representative prototype of the target class if the audio segments of the support set are affected by conspicuous background noise.

Similarly, analyzing the results by fixing *p* and varying *n* leads to uniform outputs: the AUPRC scores increase as n=(1,5,10,50) increases, with rare exceptions in the configuration with p=1. This fact confirms that the *1-shot* setting, employed for both positive and negative classes or only one of them, is not a suitable approach for classifying sound events with quick fluctuations in intensity and frequency distribution.

As expected, the scores obtained from the A3S-Synth-trained model are better than the SWC ones, followed by the US8K model outcomes. The A3S-Synth dataset provides the best performance in the combination (p,n)=(10,50) with an AUPRC score of 0.90 at channel 8, and among the several models, it benefits most from multiple support examples.

If we analyze the AUPRC values in each audio channel, there are no substantial differences between microphone positions in the same installation context. The microphones behind the license plate (positions 7–8) achieve the best performance, followed by those inside the passenger compartment (positions 1–4) and finally in the trunk (positions 5–6).

#### 5.2.2. Evaluation with Internal Labeling

In previous simulations, we tested few-shot models on data extracted from recordings acquired by eight microphones outside and inside the cockpit. The audio segments have been annotated by listening to the audio signals recorded by the sensors behind the license plate and applying the same label to all channels. In fact, audio data of microphones 7-8 show the presence of the ambulance sooner than the other positions being installed externally. We have also ascertained that they return the best performance in the ESD task, as illustrated in Table 2, Table 3 and Table 4.

We now investigate the behavior of the sensors inside the passenger compartment, focusing on the influence of cockpit sound attenuation. Because the chassis is made of soundproofing material, it acts as a barrier to the entry of the siren sound when its level is below the enclosure transmission loss at the siren tones frequencies. This fact results in a shorter duration of siren sound events in the internal recordings than in the external ones. To indirectly assess the influence of cockpit attenuation in the ESD task, we have repeated the experiments after revising the annotations only for channels 1, 2, 3, and 4. For this purpose, we have changed the labeling from siren to noise in the audio segments where the siren sound has been fully attenuated. In each audio track, the chassis soundproofing has operated differently, depending on the initial distance between source and receiver, the acquisition scenario, and the traffic noise level.

**Figure 9:**
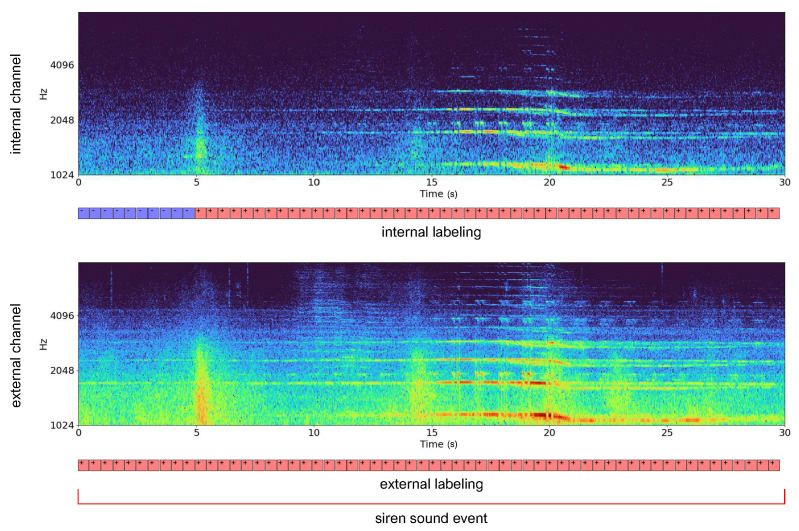
Spectrograms of siren recordings acquired by sensors inside (**top**) and outside (**bottom**) the passenger compartment with different labeling criteria for the internal channel.

After the internal labeling operation, siren instances were reduced by about 13%. Figure 9 shows an example of spectrograms of the same siren occurrence recorded by sensors inside the passenger compartment and behind the license plate. Because the siren sound in the first 5 s of the internal recording is attenuated, we attributed the noise class to this audio segment.

Table 5, Table 6 and Table 7 present testing results on channels 1-2-3-4 of the A3S-Rec dataset with internal labeling employing the SWC/US8K/A3S-Synth best prototypical models.

Considering channels 1-2-3-4, the outcomes of the recordings with internal labeling show equal or better AUPRC scores than those with external annotations in all (p,n) settings. As for the results obtained with external labeling, the few-shot configurations with (p,n)=(10,50) provide the best performance and generally show greater boosting than the *1-shot* setting. We calculated the average relative percentage increment between the external and internal labeling results: the SWC model provides the highest increase in the AUPRC score, at about 7%. This aspect shows that the good generalization capability of a model is related to the matching between the source and target domains. In fact, removing the noisiest instances from the positive class resulted in cleaner siren prototypes, more similar to the SWC ones computed from clean speech recordings.

The performance improvement with the internal labeling is correlated to the noise class attribution of uncertain siren events resulting from cockpit sound attenuation and internal car noise. Whereas the impact of the attribution of not clearly identifiable siren events to the noise class is reflected in higher scores, the algorithm exhibits delayed responsiveness in the siren recognition, as visible in Figure 9. We thus refer to the external labeling for an unbiased comparison of the effectiveness of the acquisition sensors.

We observe that the internally labeled audio data yield comparable or slightly better AUPRC scores than recordings at positions 7–8 for the experiments conducted with the SWC and US8K models. In contrast, with A3S-Synth, the external channels again outperform the internal ones. This behavior can be attributed to the similarity between the synthetic dataset generated by adding siren sounds to traffic noise recordings in the external environment and the recordings of sensors behind the license plate.

Moreover, in all models, the microphone at position 4 provides the best emergency siren detection scores. One possible reason is that the ambulances often approached the equipped car from the same direction of travel during the acquisition campaign. Thus, the alarm sound first impacted the rear, incurring less reflection from the source to the recording sensors on the back. The better response of the sensor at position 4 compared to the specular one could be due to the operator sitting near position 3, which represented an absorption surface for the incoming sound.

We have not performed tests with different labeling for audio data acquired by the microphones inside the trunk. Recordings at positions 5–6 are simultaneously affected by cabin soundproofing and mechanical component noise, so an internal labeling criterion would have resulted in an excessive reduction of siren instances.

#### 5.2.3. Evaluation across All Recordings

In the next set of experiments, we assess the few-shot techniques across all the recordings. We have identified the siren sound within individual audio tracks, so the classification task is extended over all the recordings performed by a specific sensor. If the detection in individual recordings can be interpreted as identifying the background noise perturbation produced by the siren signal, the detection across all recordings represents a generalization of the previous case. In this way, we rely on the prototypical networks for the more challenging task of discriminating a siren sound from background noise acquired in several contexts and varying both in terms of sound intensity and spectral content.

For this purpose, we have organized the instances of each audio track recorded by a given sensor in a single folder, considering channels 4, 7, and 8 as they provided the best results in the analysis within individual recordings. We construct the support set by employing the most robust (p,n) combination and evaluate the influence of increasing positive support examples. Consequently, the experiments are conducted with p∈{10,20,50} and n=50, computing AUPRC scores averaged over 10 iterations with different random support and query sets.

Table 8 presents the prototypical results across all the recordings acquired by microphones in positions 4-7-8. With a fixed n=50, a more significant number of *p* improves the scores. The best value is equal to 0.86, obtained by the A3S-Synth model in the (p,n)=(50,50) combination with data belonging to channel 7.

Despite considering the best performing audio channels and employing more instances to compute the prototypes, the detection across all recordings achieves lower scores than those within the single audio tracks. The reason can be attributed to the variability of the background noise affecting the support and query sets. Prototypes generated from spectrograms with dissimilar frequency distributions might not always enhance the features of a weak target signal, so query samples would not be distinctly associable with the positive or negative class. As confirmation of this aspect, we found that increasing the number of positive examples to p=20 in the experiments with individual recordings does not make significant contributions, achieving a maximum improvement of one percentage point. In that case, the use of only p=10 instances yields a stable representation that captures the variance of the signal in a single audio track; on the other hand, in the experiments across all recordings, the use of many support examples proves beneficial in almost all combinations. Again, the task-related model computed with the A3S-Synth dataset is the most effective.

In addition, we examine noise reduction effects by applying the harmonic-percussive source separation technique [59] to the A3S-Rec dataset. Table 9 presents prototypical results across all the recordings acquired by microphones in position 4-7-8 after harmonic filtering with a separation factor $\beta_{h}=3$. We observe an appreciable improvement provided by the filtering operations, especially for the external channels. The best AUPRC scores are attributed to the A3S-Synth model in the (p,n)={(20,50),(50,50)} combinations with data belonging to channel 8.

The results show that channels 7–8 yield better performance than channel 4. One possible explanation is that the high noise in the external recordings is easily separated and assigned to the percussive and residual components, emphasizing the harmonic siren sound. On the other hand, filtering operations in cleaner internal recordings do not improve appreciably over unfiltered audio data. We also note that using more positive examples often does not lead to better outcomes. In the filtered condition, spectrograms highlight the harmonic content of the signal, and thus, even few instances can create a representative prototype. Finally, we comment on the better scores of channel 8 compared to 7. Despite being in specular positions, the microphone at position 7 is located on the left side of the license plate. The corresponding recordings may be affected by noises with tonal components from cars in the other direction of travel, which explains the loss of performance with the filtered audio data.

### 5.3. Siren Detection with Baseline

The last experiments concern classifying the audio files belonging to the A3S-Rec dataset by using the baseline computed by the CNN described in Section 3.3.2. Training is performed with the A3S-Synth dataset as it provided the best ESD models in the previous experiments. Moreover, the use of synthetic datasets is often an effective strategy to build a representative model in the condition of scarce availability of actual data. We evaluate the CNN performance across all the recordings with and without fine-tuning for domain adaptation. For a comparison with prototypical networks, the (p,n) combinations of instances used to construct the support embeddings are employed to update the weights of the two last linear layers, as described in Section 4.2.3. The AUPRC scores are averaged over 10 different (p,n) combinations to account for the variability of the fine-tuning instances.

Table 10 presents the outcomes of the baseline model without fine-tuning tested across all the recordings of A3S-Rec (channels 4-7-8) in unfiltered and harmonic filtered conditions.

Although the results do not differ significantly, we observe the best scores for the external channels in the unfiltered conditions with an AUPRC equal to 0.65. The reason is the affinity between source and target domains, as the synthetic siren audio files have been generated simulating siren alarms immersed in urban traffic noise in the outdoor environment. On the other hand, inference on filtered data shows a slight decrease in performance at channels 7-8. Because the training was conducted on unfiltered data and the filtering accentuates any harmonic components, generic tonal sounds recorded by the external sensors may be confused with the siren alarm.

Table 11 illustrates the results of the baseline model with fine-tuning, again in unfiltered and harmonic filtered conditions.

The analysis of the fine-tuned baseline results mirrors the trend of prototypical AUPRC scores with the A3S-Synth model shown in Table 8 and Table 9. In both unfiltered and harmonic filtered conditions, many (p,n) instances for the fine-tuning improve the classification performance. Again, the effectiveness of the noise reduction technique is proven by the best results obtained with filtered data belonging to the external channels. For the internal channel, we note that fine-tuning with only 10 positive examples decreases the performance of the baseline without domain adaptation. In this case, few positive examples affected by cockpit attenuation and rapid model overfitting lead to erroneous learning of the siren class. This aspect shows an additional advantage of prototypical networks in the low-data regime. Whereas the convolutional neural network used for the baseline has been trained with few epochs to reduce the problem of overfitting on the fine-tuning data, for prototypical networks, this excessive adaptation does not affect the results due to the distance-based metrics, as investigated in [26].

In Table 12, the relative percentage increase of the few-shot achievements with respect to (CNN + fine-tuning) is presented.

In almost all cases, the most significant increments occur in the combination (p,n)=(10,50) and decrease with higher *p* values. This fact indicates that by increasing the (p,n) examples, AUPRC scores of the fine-tuned baseline approximate the few-shot outcomes. However, prototypical networks demonstrate their superior efficacy because they perform equally well with a very limited amount of support instances.

In addition, the improvement with the lowest number of data used in the (p,n)=(10,50) combination is more evident in the case of the internal microphone, meaning that the few-shot solution performs better than the (CNN + fine-tuning) when the mismatch between training and testing conditions is high.

### 5.4. Discussion

In light of the results, prototypical networks have shown to be a robust method in emergency siren detection. Similarity learning between instances and prototypes of the same class demonstrates a more effective method than learning single examples that are not always representative of the belonging class. Moreover, adopting a training dataset that matches the target domain facilitates the classification task.

However, not all few-shot techniques are as successful as prototypical networks. We have also performed experiments with relation networks [33], but they have not equaled the prototypical ones despite obtaining fair results. We attribute the motivation to the embedding generation method, considering the high noise level in our recordings. In the prototypical embedding, the noise and siren feature maps are averaged, so the noise is redistributed among all frequencies. On the other hand, in the relation embedding, the feature maps are summed element-wise, and the sum amplifies the noise representation over the siren.

In this work, our investigations have provided valuable insights for implementing in-car emergency vehicle detection systems, summarized as follows.

1. The best location for the acquisition sensor is outside the vehicle behind the license plate. This placement is not affected by wind nor cabin attenuation and gives a fast response to siren sound detection. The related disadvantage of the installation is the requirement for weatherproof sensors.
2. Because the high noise level of external recordings affects the siren detection task, a noise reduction filter such as the one proposed should be included to improve the external sensor performance.
3. Due to the effectiveness of few-shot techniques compared with traditional methods under conditions of few data and mismatch between training and test sets, the sensors inside the passenger compartment can be employed with significant deployment benefits. Although disadvantages arise from reduced responsiveness resulting from cockpit soundproofing, people talking, or the sound system, internal microphones installed in a weather-protected environment present lower costs and maintenance than the external ones.
4. The most common and dangerous situation is an ambulance approaching a car in the same direction of travel, so the most suitable microphone placement is at the rear of the vehicle. If the internal installation is chosen, interference with sound-absorbing surfaces should be avoided.

## 6. Conclusions and Future Works

In this work, we investigated a novel approach based on few-shot learning for emergency siren detection. Because our goal was to detect siren sounds acquired with recording sensors outside and inside an equipped car, collecting enough audio data to train traditional supervised deep learning models proved challenging and laborious. Thanks to few-shot strategies that require only few instances of the target domain in the learning process, we performed an exhaustive analysis of prototypical network capabilities in a real-world application. In addition, the performance of the recording sensors at different positions outside and inside the cabin provided insights for devising an emergency vehicle detection system. The experimental workflow was conducted by (i) training prototypical networks with three datasets differing in the genre and amount of audio data in several *C-way K-shot* combinations; (ii) testing each model on data from the same audio collection used in training, approaching the classification task as an open-set problem; (iii) performing emergency siren detection by applying the best prototypical models to inference on the new dataset; (iv) comparing the few-shot outcomes with a CNN baseline, with and without fine-tuning for domain adaptation; and (v) evaluating the contribution of filtering techniques for background noise reduction.

In the experiments, prototypical networks achieved promising results and outperformed a fine-tuned convolutional baseline, demonstrating superior robustness in conditions with very limited data. Our study represents the starting point for future works. From the comparative perspective, other models such as Generative Adversarial Networks could be a viable solution for creating new examples of the target class by adapting a generic training set. Possible innovations to the few-shot method could be achieved by hybrid embedding models that gain specific knowledge of the target task or by zero-shot learning techniques, which can enable the classification of new sirens without providing examples for training, but only from semantic embeddings (e.g., annotations, textual descriptions, musical notes). Finally, applicative developments can be achieved by testing the effectiveness of prototypical networks on a larger number of examples and noise contexts generated in a controlled environment such as a semianechoic chamber and deploying a framework for real-time siren detection on embedded systems.

## Figures and Tables

**Table 1:** A3S-Rec audio file composition and recording environment.

Recording	Siren (s)	Noise (s)	Location
20210506-1652	53	60	construction site
20210506-1714	19	60	coastal road
20210507-1421	38.5	60	shopping center
20210507-1426	38	60	residential suburb
20210507-1536	26	60	high-speed road
20210507-1640	25	60	city center
Total	199.5	360	

**Table 2:** A3S-Rec dataset: AUPRC of the *10-way 10-shot* SWC prototypical model tested on individual recordings for each acquisition sensor.

(*p*, *n*)	ch1	ch2	ch3	ch4	ch5	ch6	ch7	ch8
(1, 1)	0.64 ± 0.09	0.65 ± 0.08	0.66 ± 0.10	0.66 ± 0.10	0.62 ± 0.08	0.63 ± 0.09	0.68 ± 0.07	0.68 ± 0.07
(5, 1)	0.71 ± 0.11	0.69 ± 0.10	0.68 ± 0.11	0.71 ± 0.11	0.67 ± 0.11	0.67 ± 0.12	0.75 ± 0.06	0.72± 0.07
(10, 1)	0.69 ± 0.10	0.70 ± 0.10	0.69 ± 0.11	0.70 ± 0.11	0.65 ± 0.10	0.66 ± 0.10	0.75 ± 0.06	0.73 ± 0.06
(1, 5)	0.68 ± 0.11	0.66 ± 0.08	0.67 ± 0.09	0.67 ± 0.11	0.65 ± 0.13	0.67 ± 0.12	0.71 ± 0.08	0.69 ± 0.10
(5, 5)	0.72 ± 0.11	0.72± 0.10	0.73 ± 0.11	0.74 ± 0.11	0.68 ± 0.14	0.69 ± 0.13	0.77 ± 0.09	0.77 ± 0.09
(10, 5)	0.73 ± 0.12	0.73± 0.10	0.73 ± 0.12	0.75 ± 0.11	0.69 ± 0.14	0.70 ± 0.13	0.78 ± 0.09	0.76 ± 0.09
(1, 10)	0.70 ± 0.11	0.69 ± 0.10	0.69 ± 0.11	0.69 ± 0.11	0.67 ± 0.13	0.67 ± 0.13	0.71 ± 0.11	0.70 ± 0.11
(5, 10)	0.74 ± 0.11	0.74 ± 0.11	0.74 ± 0.11	0.76 ± 0.11	0.70 ± 0.14	0.71 ± 0.14	0.80 ± 0.09	0.78 ± 0.10
(10, 10)	0.74 ± 0.12	0.75 ± 0.10	0.75 ± 0.11	0.77 ± 0.10	0.70 ± 0.14	0.71 ± 0.14	0.80 ± 0.10	0.80 ± 0.10
(1, 50)	0.69 ± 0.11	0.70 ± 0.10	0.70 ± 0.10	0.71 ± 0.10	0.67 ± 0.14	0.68 ± 0.13	0.72 ± 0.09	0.72 ± 0.09
(5, 50)	0.73 ± 0.12	0.74 ± 0.11	0.74 ± 0.11	0.77 ± 0.10	0.69 ± 0.14	0.70 ± 0.13	0.81 ± 0.09	0.79 ± 0.11
(10, 50)	0.75 ± 0.11	0.75 ± 0.10	0.77 ± 0.10	0.79 ± 0.09	0.70 ± 0.14	0.73 ± 0.12	**0.82 ± 0.09**	**0.82 ± 0.10**
avg	0.71 ± 0.11	0.71 ± 0.10	0.71 ± 0.11	0.73 ± 0.10	0.67 ± 0.13	0.68 ± 0.12	0.76 ± 0.09	0.75 ± 0.09

**Table 3:** A3S-Rec dataset: AUPRC of the *10-way 10-shot* US8K prototypical model tested on individual recordings for each acquisition sensor.

(*p*, *n*)	ch1	ch2	ch3	ch4	ch5	ch6	ch7	ch8
(1, 1)	0.65 ± 0.10	0.63 ± 0.07	0.67 ± 0.09	0.65 ± 0.09	0.63 ± 0.09	0.63 ± 0.09	0.69 ± 0.07	0.68 ± 0.08
(5, 1)	0.71 ± 0.12	0.67 ± 0.09	0.69 ± 0.10	0.69 ± 0.10	0.67 ± 0.11	0.65 ± 0.11	0.74 ± 0.06	0.73 ± 0.07
(10, 1)	0.68 ± 0.11	0.68 ± 0.10	0.70 ± 0.11	0.68 ± 0.10	0.68 ± 0.10	0.67 ± 0.11	0.74 ± 0.05	0.73 ± 0.07
(1, 5)	0.67 ± 0.12	0.64 ± 0.09	0.66 ± 0.11	0.65 ± 0.14	0.64 ± 0.13	0.63 ± 0.13	0.67 ± 0.08	0.66 ± 0.10
(5, 5)	0.71 ± 0.13	0.69 ± 0.12	0.71 ± 0.14	0.69 ± 0.14	0.67 ± 0.13	0.67 ± 0.13	0.73 ± 0.08	0.73 ± 0.09
(10, 5)	0.71 ± 0.14	0.70 ± 0.12	0.72 ± 0.13	0.71 ± 0.13	0.68 ± 0.13	0.68 ± 0.13	0.74 ± 0.08	0.73 ± 0.09
(1, 10)	0.68 ± 0.14	0.67 ± 0.11	0.68 ± 0.13	0.67 ± 0.13	0.65 ± 0.13	0.66 ± 0.14	0.68 ± 0.10	0.67 ± 0.10
(5, 10)	0.72 ± 0.14	0.71 ± 0.12	0.72 ± 0.14	0.71 ± 0.14	0.68 ± 0.14	0.68 ± 0.15	0.75 ± 0.09	0.74 ± 0.10
(10, 10)	0.71 ± 0.14	0.71 ± 0.13	0.73 ± 0.14	0.72 ± 0.14	0.68 ± 0.14	0.68 ± 0.15	0.75 ± 0.09	0.75 ± 0.09
(1, 50)	0.68 ± 0.14	0.67 ± 0.11	0.68 ± 0.13	0.67 ± 0.11	0.65 ± 0.14	0.64 ± 0.13	0.69 ± 0.09	0.67 ± 0.09
(5, 50)	0.71 ± 0.13	0.72 ± 0.13	0.73 ± 0.14	0.72 ± 0.13	0.68 ± 0.15	0.68 ± 0.15	0.74 ± 0.10	0.73 ± 0.10
(10, 50)	0.73 ± 0.14	0.72 ± 0.12	0.74 ± 0.13	0.73 ± 0.14	0.70 ± 0.14	0.68 ± 0.15	**0.77 ± 0.08**	0.76 ± 0.09
avg	0.70 ± 0.13	0.68 ± 0.11	0.70 ± 0.12	0.69 ± 0.12	0.67 ± 0.13	0.66 ± 0.13	0.72 ± 0.08	0.71 ± 0.09

**Table 4:** A3S-Rec dataset: AUPRC of the *2-way 10-shot* A3S-Synth prototypical model tested on individual recordings for each acquisition sensor.

(*p*, *n*)	ch1	ch2	ch3	ch4	ch5	ch6	ch7	ch8
(1, 1)	0.63 ± 0.09	0.63 ± 0.08	0.63 ± 0.09	0.65 ± 0.11	0.61 ± 0.09	0.60 ± 0.08	0.69 ± 0.10	0.70 ± 0.10
(5, 1)	0.66 ± 0.12	0.67 ± 0.10	0.65 ± 0.11	0.68 ± 0.12	0.65 ± 0.14	0.64 ± 0.12	0.75 ± 0.12	0.76 ± 0.10
(10, 1)	0.67 ± 0.11	0.69 ± 0.09	0.68 ± 0.11	0.70 ± 0.13	0.63 ± 0.12	0.63 ± 0.10	0.75 ± 0.11	0.75 ± 0.09
(1, 5)	0.66 ± 0.11	0.67 ± 0.08	0.66 ± 0.09	0.69 ± 0.13	0.65 ± 0.13	0.65 ± 0.11	0.77 ± 0.07	0.76 ± 0.09
(5, 5)	0.72 ± 0.11	0.74 ± 0.09	0.74 ± 0.11	0.76 ± 0.12	0.69 ± 0.13	0.69 ± 0.12	0.83 ± 0.09	0.84 ± 0.07
(10, 5)	0.73 ± 0.12	0.76 ± 0.10	0.75 ± 0.12	0.78 ± 0.12	0.70 ± 0.13	0.70 ± 0.12	0.83 ± 0.08	0.84 ± 0.07
(1, 10)	0.67 ± 0.11	0.69 ± 0.09	0.69 ± 0.10	0.70 ± 0.12	0.66 ± 0.12	0.65 ± 0.12	0.77 ± 0.10	0.77 ± 0.09
(5, 10)	0.74 ± 0.11	0.76 ± 0.10	0.76 ± 0.11	0.79 ± 0.11	0.71 ± 0.13	0.70 ± 0.12	0.85 ± 0.06	0.86 ± 0.07
(10, 10)	0.75 ± 0.11	0.78 ± 0.10	0.77 ± 0.11	0.80 ± 0.12	0.71 ± 0.13	0.72 ± 0.12	0.86 ± 0.08	0.89 ± 0.05
(1, 50)	0.67 ± 0.11	0.70 ± 0.09	0.68 ± 0.08	0.71 ± 0.09	0.66 ± 0.12	0.65 ± 0.12	0.79 ± 0.09	0.79 ± 0.08
(5, 50)	0.73 ± 0.11	0.76 ± 0.11	0.76 ± 0.11	0.80 ± 0.10	0.70 ± 0.14	0.71 ± 0.12	0.87 ± 0.05	0.88 ± 0.06
(10, 50)	0.76 ± 0.11	0.79 ± 0.09	0.78 ± 0.09	0.83 ± 0.08	0.73 ± 0.13	0.74 ± 0.10	0.89 ± 0.05	**0.90 ± 0.05**
avg	0.70 ± 0.11	0.72 ± 0.09	0.71 ± 0.10	0.74 ± 0.11	0.68 ± 0.13	0.67 ± 0.11	0.80 ± 0.08	0.81 ± 0.08

**Table 5:** A3S-Rec dataset: AUPRC of the *10-way 10-shot* SWC prototypical model, considering internal labeling for the recordings of channels 1-2-3-4.

(*p*, *n*)	ch1	ch2	ch3	ch4
(1, 1)	0.71 ± 0.09	0.71 ± 0.08	0.69 ± 0.10	0.71 ± 0.09
(5, 1)	0.74 ± 0.09	0.73 ± 0.10	0.75 ± 0.10	0.75 ± 0.07
(10, 1)	0.75 ± 0.09	0.76 ± 0.08	0.75 ± 0.10	0.75 ± 0.09
(1, 5)	0.71 ± 0.11	0.71 ± 0.10	0.69 ± 0.10	0.75 ± 0.09
(5, 5)	0.79 ± 0.09	0.78 ± 0.08	0.79 ± 0.10	0.79 ± 0.09
(10, 5)	0.79 ± 0.08	0.79 ± 0.08	0.79 ± 0.09	0.80 ± 0.09
(1, 10)	0.72 ± 0.10	0.72 ± 0.10	0.70 ± 0.09	0.75 ± 0.09
(5, 10)	0.79 ± 0.09	0.78 ± 0.09	0.79 ± 0.09	0.80 ± 0.09
(10, 10)	0.81 ± 0.07	0.82 ± 0.07	0.81 ± 0.08	0.82 ± 0.08
(1, 50)	0.71 ± 0.10	0.72 ± 0.09	0.71 ± 0.10	0.77 ± 0.09
(5, 50)	0.80 ± 0.08	0.80 ± 0.08	0.80 ± 0.09	0.81 ± 0.09
(10, 50)	0.81 ± 0.07	**0.82 ± 0.07**	0.81 ± 0.08	**0.82 ± 0.10**
avg	0.76 ± 0.09	0.76 ± 0.08	0.76 ± 0.09	0.78 ± 0.09

**Table 6:** A3S-Rec dataset: AUPRC of the *10-way 10-shot* US8K prototypical model, considering internal labeling for the recordings of channels 1-2-3-4.

(*p*, *n*)	ch1	ch2	ch3	ch4
(1, 1)	0.69 ± 0.12	0.67 ± 0.10	0.67 ± 0.11	0.68 ± 0.09
(5, 1)	0.72 ± 0.13	0.68 ± 0.10	0.70 ± 0.12	0.72 ± 0.11
(10, 1)	0.70 ± 0.13	0.69 ± 0.10	0.70 ± 0.12	0.72 ± 0.11
(1, 5)	0.71 ± 0.14	0.68 ± 0.13	0.68 ± 0.11	0.69 ± 0.11
(5, 5)	0.72 ± 0.13	0.72 ± 0.14	0.73 ± 0.13	0.76 ± 0.12
(10, 5)	0.75 ± 0.14	0.73 ± 0.14	0.75 ± 0.13	0.77 ± 0.12
(1, 10)	0.70 ± 0.14	0.69 ± 0.13	0.69 ± 0.14	0.69 ± 0.12
(5, 10)	0.74 ± 0.15	0.72 ± 0.16	0.74 ± 0.14	0.76 ± 0.12
(10, 10)	0.76 ± 0.13	0.74 ± 0.15	0.76 ± 0.13	0.78 ± 0.12
(1, 50)	0.70 ± 0.14	0.68 ± 0.13	0.69 ± 0.13	0.70 ± 0.12
(5, 50)	0.75 ± 0.14	0.72 ± 0.15	0.75 ± 0.14	0.78 ± 0.12
(10, 50)	0.77 ± 0.14	0.74 ± 0.15	0.76 ± 0.14	**0.79 ± 0.11**
avg	0.73 ± 0.14	0.71 ± 0.13	0.72 ± 0.13	0.74 ± 0.11

**Table 7:** A3S-Rec dataset: AUPRC of the *2-way 10-shot* A3S-Synth prototypical model, considering internal labeling for the recordings of channels 1-2-3-4.

(*p*, *n*)	ch1	ch2	ch3	ch4
(1, 1)	0.66 ± 0.10	0.65 ± 0.08	0.65 ± 0.12	0.68 ± 0.11
(5, 1)	0.70 ± 0.12	0.68 ± 0.11	0.72 ± 0.10	0.75 ± 0.10
(10, 1)	0.70 ± 0.13	0.69 ± 0.10	0.71 ± 0.11	0.75 ± 0.11
(1, 5)	0.69 ± 0.11	0.68 ± 0.08	0.67 ± 0.10	0.70 ± 0.10
(5, 5)	0.76 ± 0.12	0.77 ± 0.09	0.76 ± 0.10	0.80 ± 0.10
(10, 5)	0.77 ± 0.11	0.77 ± 0.09	0.78 ± 0.10	0.81 ± 0.09
(1, 10)	0.69 ± 0.11	0.69 ± 0.07	0.69 ± 0.09	0.71 ± 0.09
(5, 10)	0.77 ± 0.11	0.78 ± 0.08	0.78 ± 0.10	0.81 ± 0.09
(10, 10)	0.79 ± 0.11	0.81 ± 0.07	0.80 ± 0.08	0.82 ± 0.09
(1, 50)	0.69 ± 0.11	0.69 ± 0.10	0.70 ± 0.10	0.71 ± 0.10
(5, 50)	0.77 ± 0.12	0.79 ± 0.09	0.79 ± 0.10	0.81 ± 0.10
(10, 50)	0.80 ± 0.10	0.81 ± 0.08	0.80 ± 0.09	**0.83 ± 0.10**
avg	0.73 ± 0.11	0.74 ± 0.09	0.74 ± 0.10	0.77 ± 0.10

**Table 8:** A3S-Rec dataset: AUPRC of the best prototypical models across all the recordings of channels 4-7-8.

	SWC			US8K			A3S-Synth		
**(** ***p*** **,** ***n*** **)**	**ch4**	**ch7**	**ch8**	**ch4**	**ch7**	**ch8**	**ch4**	**ch7**	**ch8**
(10, 50)	0.59 ± 0.05	0.72 ± 0.07	0.76 ± 0.04	0.59 ± 0.05	0.65 ± 0.03	0.66 ± 0.03	0.67 ± 0.03	0.82 ± 0.02	0.82 ± 0.02
(20, 50)	0.62 ± 0.05	0.77 ± 0.02	0.78 ± 0.03	0.62 ± 0.04	0.67 ± 0.02	0.69 ± 0.02	0.73 ± 0.05	0.84 ± 0.02	0.83 ± 0.03
(50, 50)	0.64 ± 0.05	0.81 ± 0.03	0.80 ± 0.02	0.63 ± 0.02	0.67 ± 0.01	0.69 ± 0.01	0.73 ± 0.03	**0.86 ± 0.02**	0.85 ± 0.02

**Table 9:** A3S-Rec dataset: performance of the best prototypical models across all the recordings of channels 4-7-8 with harmonic filter.

	SWC			US8K			A3S-Synth		
**(** ***p*** **,** ***n*** **)**	**ch4**	**ch7**	**ch8**	**ch4**	**ch7**	**ch8**	**ch4**	**ch7**	**ch8**
(10, 50)	0.67 ± 0.05	0.86 ± 0.01	0.88 ± 0.01	0.60 ± 0.05	0.76 ± 0.02	0.80 ± 0.03	0.70 ± 0.04	0.85 ± 0.02	0.90 ± 0.01
(20, 50)	0.71 ± 0.02	0.86 ± 0.01	0.87 ± 0.01	0.62 ± 0.04	0.78 ± 0.02	0.82 ± 0.02	0.75 ± 0.02	0.87 ± 0.01	**0.91 ± 0.01**
(50, 50)	0.71 ± 0.02	0.86 ± 0.00	0.88 ± 0.01	0.62 ± 0.03	0.78 ± 0.02	0.83 ± 0.02	0.75 ± 0.02	0.86 ± 0.01	**0.91 ± 0.00**

**Table 10:** A3S-Rec dataset: AUPRC of the baseline model without fine-tuning for ESD across all the recordings of channels 4-7-8.

Filtering	ch4	ch7	ch8
no	0.60	**0.65**	**0.65**
harmonic	0.62	0.64	0.64

**Table 11:** A3S-Rec dataset: AUPRC of the baseline model with fine-tuning in several (p,n) combinations for ESD across all the recordings of channels 4-7-8.

Filtering	(*p, n*)	ch4	ch7	ch8
	(10,50)	0.45 ± 0.07	0.78 ± 0.04	0.80 ± 0.02
no	(20,50)	0.65 ± 0.03	0.81 ± 0.01	0.81 ± 0.01
	(50,50)	0.70 ± 0.03	**0.84 ± 0.02**	0.83 ± 0.01
	(10,50)	0.57 ± 0.07	0.82 ± 0.01	0.84 ± 0.02
harmonic	(20,50)	0.71 ± 0.04	0.83 ± 0.01	0.86 ± 0.01
	(50,50)	0.73 ± 0.03	0.85 ± 0.01	**0.88 ± 0.02**

**Table 12:** A3S-Rec dataset: AUPRC relative percentage increase from fine-tuned baseline to best prototypical score across all the recordings of channels 4-7-8.

Filtering	(*p, n*)	ch4	ch7	ch8
	(10, 50)	48.8%	5.7%	3.0%
no	(20, 50)	12.8%	4.6%	2.6%
	(50, 50)	4.4%	1.8%	2.0%
	(10, 50)	21.6%	4.1%	6.6%
harmonic	(20, 50)	6.0%	4.3%	4.8%
	(50, 50)	2.5%	1.0%	4.0%

## Data Availability

The A3S-Synth and A3S-Rec datasets are available online at https://github.com/michelacantarini/Few-Shot-Emergency-Siren-Detection (accessed on 5 June 2022).
